# Disparities in eye clinic patient encounters among patients requiring language interpreter services

**DOI:** 10.1186/s12886-022-02756-6

**Published:** 2023-03-02

**Authors:** Lucy I. Mudie, Jennifer L. Patnaik, Zafar Gill, Marissa Wagner, Karen L. Christopher, Leonard K. Seibold, Cristos Ifantides

**Affiliations:** 1 Department of Ophthalmology, University of Colorado, 1675 Aurora Court F731, Aurora, CO 80045 USA; 2 Department of Surgery, Denver Health Medical Center, 660 Bannock Street, Denver, CO 80204 USA

**Keywords:** Medical interpreters, Limited english proficiency patients, Ophthalmology

## Abstract

**Background:**

Communication barriers are a major cause of health disparities for patients with limited English proficiency (LEP). Medical interpreters play an important role in bridging this gap, however the impact of interpreters on outpatient eye center visits has not been studied. We aimed to evaluate the differences in length of eyecare visits between LEP patients self-identifying as requiring a medical interpreter and English speakers at a tertiary, safety-net hospital in the United States.

**Methods:**

A retrospective review of patient encounter metrics collected by our electronic medical record was conducted for all visits between January 1, 2016 and March 13, 2020. Patient demographics, primary language spoken, self-identified need for interpreter and encounter characteristics including new patient status, patient time waiting for providers and time in room were collected. We compared visit times by patient’s self-identification of need for an interpreter, with our main outcomes being time spent with ophthalmic technician, time spent with eyecare provider, and time waiting for eyecare provider. Interpreter services at our hospital are typically remote (via phone or video).

**Results:**

A total of 87,157 patient encounters were analyzed, of which 26,443 (30.3%) involved LEP patients identifying as requiring an interpreter. After adjusting for patient age at visit, new patient status, physician status (attending or resident), and repeated patient visits, there was no difference in the length of time spent with technician or physician, or time spent waiting for physician, between English speakers and patients identifying as needing an interpreter. Patients who self-identified as requiring an interpreter were more likely to have an after-visit summary printed for them, and were also more likely to keep their appointment once it was made when compared to English speakers.

**Conclusions:**

Encounters with LEP patients who identify as requiring an interpreter were expected to be longer than those who did not indicate need for an interpreter, however we found that there was no difference in the length of time spent with technician or physician. This suggests providers may adjust their communication strategy during encounters with LEP patients identifying as needing an interpreter. Eyecare providers must be aware of this to prevent negative impacts on patient care. Equally important, healthcare systems should consider ways to prevent unreimbursed extra time from being a financial disincentive for seeing patients who request interpreter services.

## Background

It has long been known that racial and ethnic disparities exist in access to medical care [1–3]. The Agency for Healthcare Research and Quality (AHRQ) reports annually on healthcare disparities. AHRQ data from 2019 showed racial and ethnic minorities receive worse care than white patients for 33 to 40% of quality measures (which includes private insurance coverage, access to specialist medical care, and receiving routine preventative care such as influenza vaccine and pap smears) [4]. Although many quality measures have improved over the past two decades, disparities persist, and for some the gap has widened [4]. Among the many reasons for disparities in healthcare, communication barriers, often secondary to limited English proficiency (LEP), are high on the list [3]. Language barriers can lead to poor understanding of diagnoses, poor treatment alliance between the patient and provider, and suboptimal care with poorer health outcomes [5]. LEP can trigger cognitive bias by providers, and may deter patients from presenting for help in a timely manner. In a study of migrant workers, perceived lack of interpreter was the number one barrier to accessing health care [6]. Prior studies of Emergency Room (ER) visits have reported that patients who don’t speak English are 24% more likely to have an unplanned second ER visit within 3 days [7], and in one study their average cost of an ER visit was around $40 more [8].

If the language barrier is eased, many care disparities can also be reduced; for example, Jacobs et al. [9] reported a cost saving of $100/visit if the treating ER physician was bilingual in English and Spanish. Another study found that Hispanics who spoke English received the same care as non-Hispanic English speakers [10]. Certified interpreters have been suggested as a way to overcome language barriers, however their use varies dramatically. Blay et al. [11] reported variation in the use of interpreters from 16 to 71% depending on the hospital setting. The often cited reasons for not using a formal interpreter service are lack of availability, perceived time or budget constraints, or a lack of training in the use of interpreters [11]. Even if an interpreter is used, some studies suggests that practitioners and interpreters experience difficulties in their collaboration such as cross-cultural translation, emotional and interpersonal challenges, all of which can negatively affect services to patients with LEP [12].

In ophthalmology, high-risk factors for eye disease and/or vision loss that have consistently been identified include increasing age, racial/ethnic minority, and low socioeconomic status [13]. Individuals cite trust, communication, and cost/lack of insurance as major barriers to accessing eye care [14, 15]. In one patient focus group, 20% of the barriers to eyecare were comments on poor interactions with the eyecare provider due to communication failures [13]. In a study of a glaucoma clinic at a safety-net hospital in San Francisco, USA, difficulties related to medial interpretation made up 23% of the barriers to follow up care [16]. The same study suggested Latinos and Asian-Pacific Islanders were particularly affected by difficulties related to medical interpretation and long waiting times in the clinic [16]. Although the literature to date highlights the importance of communication and emphasizes LEP as a barrier to eyecare, our understanding of the influence of medical interpreters has not been well studied. For example, although some may assume encounters where an interpreter is used with LEP patients are longer than encounters with primary English speakers, this has not been proven. Likewise, it is unknown if providers may change their practice patterns when using a medical interpreter, such as more formally reviewing medication lists and allergies. As the foreign-born population of the United States is expected to grow, providers will continue to take care of LEP patients for the foreseeable future, thus it is vital for ophthalmologists to understand the impact that medical interpreters may have on their practice. In order to begin to understand the influence of interpreters on the eyecare visits, we undertook a retrospective review of data from patient encounters at the Denver Health Eye Clinic, comparing characteristics of encounters where patients self-identified as requiring an interpreter to encounters with English speakers. Specifically, we examined the length of time spent with technician and physician, time waiting for physician, as well as whether medication lists, problem lists, and allergies were reviewed, and after visit summaries were printed.

## Methods

The study received approval from the Colorado Multiple Institutional Review Boards and was conducted in accordance with the tenets set forth by the Declaration of Helsinki. A retrospective review of the characteristics of all patient encounters between January 1, 2016 to March 13, 2020 was conducted for the eye clinic of a safety-net hospital, Denver Health. Using Epic® (Verona, WI) electronic medical record (EMR), the “SlicerDicer” feature was used to generate reports of various patient or encounter features. We first selected all encounters within the eye clinic for our specified dates (1/1/2016 through 3/13/2020 to exclude the COVID-19 period) as our base “population”, then selected “interpreter needed” as our “slice”, then we selected our variables of interest as “measures”, specifically we selected: patient age at visit, new patient status, primary language spoken, encounter provider, primary financial class, no-show probability, time with technician, time with provider, time waiting for provider, review of allergies, medications, and problem list, and whether the after visit summary (AVS) was printed. The data was then exported at the visit-level from SlicerDicer, patient names and addresses were removed, and the data was securely transferred to statistical software for analysis.

Denver Health Medical Center is a large level-one trauma center and safety-net hospital in Denver, Colorado which provides emergency, primary and specialty care to all Denver residents, regardless of their ability to pay. The hospital sees a disproportionate share of Denver’s LEP patients, lower-socioeconomic and vulnerable populations. In 2018, Denver Health had almost 1 million patient visits; of these, 460,000 were visits by Hispanic patients, 271,000 were White/Caucasian patients, and 140,000 were Black patients [17]. The Denver Health Eye Clinic primarily uses phone or video interpreter services. For Spanish language services, there are dedicated Denver Health phone interpreters available during business hours, and for other languages, an independent translation service is utilized. If the lines to the dedicated Denver Health Spanish interpreters are busy, providers are redirected to the contracted provider. The majority of the front desk staff and ophthalmic technicians are bilingual in English and Spanish, and many of the providers have some proficiency in Spanish, however none of our eyecare providers are certified in medical Spanish to provide healthcare services. The “interpreter needed” variable used in our analysis is a patient level variable tracked in the EMR. It is typically collected by our administrative staff at the time of patient registration and patients may self-identify as needing an interpreter. Review of allergies, medication and problem list is typically part of our technician workflow, although physicians are also encouraged to review this during the visit. “No-show probability” is a variable generated by SlicerDicer for an individual patient based on all their outpatient appointments in our system. The time with technician and time waiting for physician variables are based on timestamps manually created by our technicians (the technicians mark when they begin working up a patient and once their work up is complete, they mark the patient as ready and waiting for the physician). The time with physician variable is collected from an automatic time stamp when the physician opens the chart in the exam room until the patient checks out (automatic time stamp) or physician marks the chart complete (manual time stamp). Our clinic does not use scribes, and if a patient is seen by multiple physicians, then total time any physician spends with the chart open in the exam room is recorded.

### Statistical analysis

Descriptive statistics were used to report number of total visits by preferred language and self-identified need for an interpreter. We excluded from analysis patients who had English listed as their primary language spoken as well as “interpreter required” (*n* = 1,206), as this could include interpreter for hearing impairment. We also excluded patients with missing status of interpreter (*n* = 241) and unknown language (*n* = 820), and we excluded any visit with an encounter of 0 min as this could include encounters other than face-to-face visits, such as encounter for prescription refill or an encounter for telephone call.

Since patients could have multiple encounters, patient-level data was obtained from the patient’s first visit. Patient age at first visit and standard deviation were presented for five groups: preferred language of English, preferred language of Spanish with and without “interpreter needed”, and preferred other languages with and without “interpreter needed”. Percentages of no-show probability and primary financial class were also presented for these five patient groups. Age and no-show probability were not normally distributed therefore groups were compared with the Wilcoxon rank sum test.

Total visits were also presented by the same five patient groups. Percentages were used to present categorical outcomes of interest: new patient visits, AVS printed, allergies reviewed, problem list reviewed, and medication list reviewed. Continuous outcomes were described by least squares (LS) means and standard errors for time with technician, time waiting for physician, and time with physician. Comparisons across groups were performed with proc genmod linear and logistic regression modeling with generalized estimating equations to account for the correlation of repeated visits for patients.

The top five preferred languages other than English (Spanish, Arabic, Amharic, Vietnamese, and Russian) were further analyzed to assess if self-identification of need for an interpreter impacted patient time. Patient times included time waiting to be roomed, time with technician, time waiting for physician, and time with physician. Modeling was performed to account for patients having repeat visits and to adjust for age at visit and new patient visit. In addition, time with physician was also adjusted for attending physician as a fixed effect. Self-identification of needing an interpreter was compared to English speaking patients and to speakers of the same language who did not identify as needing an interpreter for each of the five patient language groups separately.

A *p*-value < 0.01 was considered statistically significant, and all analysis was conducted using SAS version 9.4 (Cary, North Carolina, USA).

## Results

A total of 87,157 patient encounters of 36,503 unique patients occurred during our study period. Most patients spoke English as a primary language and the most common languages other than English were (in order of frequency): Spanish, Arabic, Amharic, Vietnamese, Russian, Nepali, Tigrinya, French, and Somali (Table 1). For encounters where English was not the primary language spoken by the patient, 79.0% (26,443/33,493) self-identified as requiring an interpreter. The percentage of visits where an interpreter was needed by language spoken is shown in Table 1. Table 2 compares patient demographics by language spoken and self-identified need for an interpreter, while Table 3 compares encounter characteristics by language and self-identified need for an interpreter. The average age at visit was between 30 and 50 years for all groups, with younger patients being less likely to identify as requiring an interpreter than older patients.

**Table 1:** Language Characteristics of Patient Encounters, April 2016-March 13, 2020

	Visits n	Self-identified as interpreter needed n (%)
Total Visits	87,157	26,443 (30.3%)
Number of visits by language:		
English	53,664	0
Spanish	25,997	21,143 (81.3%)
Arabic	1,287	832 (64.6%)
Amharic	929	472 (50.8%)
Vietnamese	659	515 (78.1%)
Russian	647	532 (82.2%)
Nepali	470	354 (75.3%)
Tigrinya	332	243 (73.2%)
French	294	175 (59.5%)
Somali	243	165 (67.9%)
Other	2,635	2,012 (76.4%)

**Table 2:** Patient characteristics by language grouping and self-identified need for an interpreter

	English	Spanish		Other Languages	
*Self-identified as interpreter needed?*	*N/A*	*No*	*Yes*	*No*	*Yes*
Unique patients, *n*	23,309	2,366	7,967	995	1,866
Average (SD) age at first visit in years	40.8 (21.6)	31.9^a^ (23.2)	41.4^b^ (24.2)	35.9^a^ (24.7)	49.4^a,b^ (24.2)
%No show probability	27.7%	18.9%^a^	14.9%^a,b^	19.0%^a^	15.0%^a,b^
%Primary Financial Class^c^					
*Commercial*	12.2%	4.8%	3.0%	6.4%	1.7%
*Correctional Care*	2.6%	0.7%	0.1%	0.1%	0.1%
*Fin Assist*	2.5%	21.8%	34.4%	17.9%	23.5%
*Medicaid*	49.4%	54.7%	35.8%	62.8%	49.3%
*Medicare*	33.1%	17.0%	26.4%	12.8%	25.3%
*Workers Comp*	0.2%	1.0%	0.2%	0%	0%

*Abbreviations*: *SD* Standard Deviation, *Fin* Financial

^a^Significantly different compared to English *p* < 0.01, Wilcoxon rank sum test

^b^Significantly different compared to no interpreter of the same language group, *p* < 0.01, Wilcoxon rank sum test

^c^Missing for 814 records. Not tested for statistical comparisons

**Table 3:** Encounter characteristics by language grouping and self-identified need for an interpreter

	English	Spanish		Other Languages	
*Self-identified as interpreter needed?*	*N/A*	*No*	*Yes*	*No*	*Yes*
Total Visits	53,664	4,854	21,143	2,198	5,303
%New patient visits	41.2%	46.5%^a^	35.8%^a,b^	43.4%	33.3%^a,b^
LS Means (SE) time with technician in minutes	20.0 (0.1)	19.0 (0.3)^a^	19.8 (0.2)	19.6 (0.6)	21.5(0.4)^a^
LS Means (SE) time waiting for physician in minutes	15.5 (0.1)	16.2 (0.3)	15.6 (0.2)	15.4 (0.5)	14.7 (0.3)
LS Means (SE) time with physician in minutes	18.7 (0.2)	17.6 (0.5)	18.2 (0.2)	18.2 (0.8)	19.5 (0.5)
%AVS printed	65.2%	63.4%	66.9%^a,b^	64.6%	68.4%^a,b^
%Allergies Reviewed	96.0%	97.0%^a^	96.3%	96.4%	95.2%^a^
%Problem List Reviewed	47.8%	45.0%^a^	48.5%^b^	44.4%^a^	47.7%
%Medication List Reviewed	96.7%	97.6%^a^	96.8^b^	97.4%	96.1%

*Abbreviations: LS* Least Squares, *SE* Standard Error, *AVS* After Visit Summary

^a^Significantly different compared to English *p* < 0.01

^b^Significantly different compared to no interpreter of the same language group, *p* < 0.01

Patients with a primary language other than English were less likely to “no-show” to their appointment than English speakers, regardless of whether they needed an interpreter or not, and patients identifying as needing an interpreter were even less likely to “no-show” than their same language counterparts (Table 2). The average time with ophthalmic technician was almost two minutes longer for patients identifying as requiring an interpreter whose primary language was not English or Spanish (Table 3). There were no differences in time waiting for physician or time spent with physician between the language groups. Patients identifying as requiring an interpreter were more likely to have their After Visit Summary (AVS) printed for them at the end of the visit compared to both English speakers and to the same language group with no interpreter needed.

The results of our linear regression models are shown in Tables 4 with English speakers as the reference group and Table 5 with speakers of the same language who did not identify as needing an interpreter as the reference group. After adjusting for age at visit, new patient status, whether the physician was an attending or resident, and repeat visits of the same patient, we found that there were no significant differences in time with technician or physician, or in time waiting to be roomed by technician or time waiting for physician.

**Table 4:** Adjusted impact on time aspects of encounter for patients self-identifying as needing an interpreter compared to English speakers

Adjusted^a^ change in time (minutes) for interpreter needed vs English speakers	Spanish β (SE)	Arabic β (SE)	Amharic β (SE)	Vietnamese β (SE)	Russian β (SE)
# Patient visits self-identifying as interpreter needed	21,143	832	472	515	532
# Patient visits no interpreter needed	4,854	455	457	144	115
Time waiting to be roomed	-0.2 (0.2)	-0.5 (1.1)	2.5 (1.4)	-0.4 (1.5)	2.8 (1.3)
Time with technician	-0.2 (0.2)	1.1 (1.0)	0.7 (1.0)	3.1 (2.3)	3.5 (1.8)
Time waiting for physician	0.1 (0.2)	-2.3 (0.6)	0.3 (0.9)	-0.1 (1.5)	0.4 (1.7)
Time with physician	-0.4 (0.3)	2.2 (1.4)	-1.3 (1.4)	-0.1 (1.5)	-0.8 (1.4)

Number of English speaker patient visits is 53,664

*Abbreviations*: *SE* Standard Error

^a^Adjusted for repeated patient visits, age at visit, new patient visit. Time with physician also adjusted for attending physician

**Table 5:** Adjusted impact on time aspects of encounter for patients self-identifying as needing for an interpreter compared to speakers of the same language who did not identify as needing an interpreter

Adjusted^a^ change in time (minutes) for interpreter needed vs no interpreter needed for speakers of the same language	Spanish β (SE)	Arabic β (SE)	Amharic β (SE)	Vietnamese β (SE)	Russian β (SE)
# Patient visits self-identifying as interpreter needed	21,143	832	472	515	532
# Patient visits no interpreter needed	4,854	455	457	144	115
Time waiting to be roomed	0.7 (0.4)	-0.6 (1.6)	2.9 (1.8)	3.4 (2.3)	3.5 (3.1)
Time with technician	0.5 (0.4)	0.6 (1.9)	3.1 (1.3)	1.2 (3.3)	2.9 (2.3)
Time waiting for physician	-0.3 (0.4)	-1.1 (1.1)	-0.5 (1.4)	0.1 (1.9)	2.6 (2.2)
Time with physician	0.2 (0.6)	2.1 (2.4)	1.9 (1.8)	-2.4 (3.6)	1.2 (5.0)

*Abbreviations*: *SE* Standard Error

^a^Adjusted for repeated patient visits, age at visit and new patient visit. Time with physician also adjusted for attending physician

## Discussion

Our study presents several important findings as the first paper to examine the differences in the length of eyecare visits between English speakers and LEP patients who self-identify as requiring a medical interpreter. We found that the self-identification of need for an interpreter had greater impact on time with technician than time with provider; this is intuitive since our technicians are required to collect more history. The technicians are also often responsible for the initial refraction, which can be difficult even for English speakers. In this clinic, which sees many vulnerable populations, poor literacy may further contribute to this language barrier, making refraction as well as medical interpretation even more challenging. Unfortunately, literacy status is not routinely recorded in the EMR so this study could not adjust for this, which may have influenced the results.

Although patients who identified as needing an interpreter for languages other than Spanish were shown to spend more time with technician in unadjusted analyses, after we adjusted for repeated patient visits, age at visit and new patient status, this did not reach statistical significance. The longer time could be accounted for by time waiting for an interpreter to be available, time for interpretation itself, and/or adjustments in communication strategies and behavior. The possibility of adjustment in communication strategy requires further investigation. If the provider was presenting the same information to all groups, we would expect that the encounters with an interpreter take longer to allow for translation of the information. Further studies could evaluate this by observing the same encounter such as a cataract pre-operative visit for patients with different language preference and including the need for interpreters.

We also found that there was no difference in time waiting for technician or provider for any language group. Long wait times are often cited as barriers for LEP patients seeking care [16]. One hypothesis for long wait times could be that LEP patients are not prioritized in the waiting room since staff may perceive their visit to be more difficult or take longer. However, our data contradicts this, and suggests that if waiting room times are long, they are experienced equally by all patients in the clinic.

This study found that LEP patients were more likely to keep an appointment once it had been made. It is possible this reflects lack of interpreter use by front desk staff. For example, patients may not want to call again once an appointment has been made. Another possible explanation is that LEP patients were more likely to receive their After Visit Summary (AVS) than English speakers, and this AVS includes their next appointment time. Prior studies have suggested even when interpreters are used in a clinic by nursing staff and providers, patients will often try and “get by” at the front desk without an interpreter [18]. Like our technicians, almost all of our clinic schedulers are bilingual in Spanish and English. However, this does not help patients who speak a language other than Spanish or English, and the effect was seen for both Spanish and other languages. This suggests that there may be other factors contributing, such as value placed on the appointment or cultural differences.

### Limitations

Our study has several limitations. First, patients self-identify their primary language and whether they need an interpreter or not. In our study, almost one quarter of patients whose primary language was not English self-identified as proficient in English and not requiring an interpreter. This could result an underestimation of effect if patients over-estimate their proficiency in English and falsely identify as not requiring an interpreter to avoid perceived bias. Additionally, if a patient falsely denies need for an interpreter, it raises the question of whether they truly understand the details of all discussions, such as the risks of surgery, but are embarrassed to admit this lack of understanding, which could adversely affect quality of care. Alternatively, patients sometimes come to their appointments with English-speaking relatives or friends, and prefer to have their companion translate for them, which is not recorded typically. Prior studies have reported that interpreter utilization changes with how they are offered: "In what language do you prefer to receive your medical care?" appears to be mostly likely to result in appropriate interpreter utilization [19]. Our EMR only records whether an interpreter was self-identified as needed, we cannot to be sure that a qualified medical interpreter was used for the entire visit in every case where it was needed. Further, these results highlight the impact of primarily remote interpreters as are found in our clinic, and results may be different to in person interpreters. Although one study during the COVID-19 pandemic reported no difference between remote and in person interpreters [20], it has not been widely examined.

In our study, time with technician, time with physician, and time waiting for physician, were variables generated from both automatic and manual timestamps in the patient’s encounter. Using this audit log data eliminates the Hawthorne effect [21], however it allows for imprecision in the measurement since, for example, some technicians or physicians may be quicker at opening the chart once they enter an exam room, or others may open a chart in the room, but then become engaged in other activities. Differences in practice are somewhat addressed by our large sample size with many different providers, so that our results reflect real-world variations in clinic-flow. A prior study in ophthalmology clinics found audit log data were within three minutes of manually observed time-motion data [22]. Our study did not collect any directly observed time-motion data to validate the audit log data as our main outcome was the relative difference between the different groups of patients, however it would be of interest in future studies to perform such validation measurements. There are also limitations of using vendor-generated audit log data, specifically a limited ability for outside researchers to replicate findings and in generalizability of results [23]. Audit log data has been used in ophthalmology to evaluate the impact of trainees on appointment length [24], and to report how ophthalmologists spend their time using electronic health records [25]. Other specialties have proposed user-event log data as valid means of assessing the impact of organization changes [26], and the vendor-generated data from SlicerDicer has been used to track documentation for quality metrics in pediatric asthma [27], compare PET/CT to endoscopy for detection of oropharyngeal carcinoma [28], as well to report the disposition of patients admitted with COVID-19 [29]. Although no other publications have used SlicerDicer to report on length of time of eyecare visits, our novel method has given us a unique perspective on encounters with LEP patients.

Finally, most of our technicians are bilingual in English and Spanish, which could have impacted our results for time with technician for Spanish speakers. The data collection method we used does not specify which technician took part in the encounter and so we are unable to account for bilingual staff. This is an important area for future studies as prior reports suggest significant cost savings with bilingual staff [9], and it is likely to impact time as well.

In addition to understanding the impact of bilingual staff, our study raises the question of whether the same care is being delivered if the visit is the same length of time regardless of whether an interpreter is used. According to the interpreter service contracted by our hospital it takes an average of 16 s to get an interpreter on the line [30], and as vendor reported data this is likely a generous estimate. Nonetheless, it seems unlikely that the provider is calling an interpreter and communicating the same amount of information to the patient through an interpreter without using any extra time. This an important point that future studies must evaluate. Further, it is still to be determined whether this difference is associated with patient outcomes or satisfaction, and these are significant questions that should be targeted by future research.

## Conclusions

Overall, our study suggests there are discrepancies between encounters with and without an interpreter that are unlikely to be explained by interpretation time alone. It appears providers may adjust their communication strategies when patients self-identify as requiring an interpreter. Sometimes this may be beneficial, such as being more likely to provide written instructions in an after-visit summary, however adjusting communication strategies to aim for similar appointment lengths may lower the standard of care delivered to patients requiring an interpreter. Although health care organizations that receive federal funding are mandated to provide language services to LEP patients [31], the US healthcare system does not specifically provide additional resources to hospitals and practices to care for LEP patients. This puts the financial burden of any additional unreimbursed time with LEP patients on hospitals and practices. This could create a financial disincentive to spend additional time with LEP patients. Our hope is that these data can be used to spur difficult, yet necessary, conversations with all stakeholders regarding expectations and resources for LEP patients with the goal of informing future policy interventions. There is hope for future change. The Centers for Medicare & Medicaid Services (CMS) conducted a listening session series between 2020 and 2022 with stakeholders who are driving health equity across all CMS programs. They received feedback related to numerous topics, one of which was opportunities in reimbursement and benefit design related to language barriers [32]. This undertaking is still ongoing. For the time being, providers must be conscious of adjusted behaviors and communication strategies for LEP patients and ensure it does not negatively impact patient care. As the cultural and linguistic diversity of the United States continues to grow, engaging our health care system to deliver care effectively across language barriers is an essential investment in our future.

## Data Availability

The data that support the findings of this study are available from the corresponding author but restrictions apply to the availability of these data, which were used under license for the current study, and so are not publicly available. Data are however available from the authors upon reasonable request.

## Abbreviations

LEP: Limited English Proficiency
AHRQ: Agency for Healthcare Research and Quality
ER: Emergency Room
EMR: Electronic Medical Record
AVS: After Visit Summary
LS: Least Squares
CMS: Centers for Medicare & Medicaid Services
